# Adhesion of antibiotic-resistant bacteria to patient-applied thermoplastic medical devices

**DOI:** 10.1017/ash.2026.10367

**Published:** 2026-04-20

**Authors:** Catherine Brock, Dev Mehta, Terrence Ravine

**Affiliations:** 1 Occupational Therapy, Pat Capps Covey College of Allied Health Professions, University of South Alabama, Mobile, USA; 2 Biomedical Sciences, Pat Capps Covey College of Allied Health Professions, University of South Alabama, Mobile, USA

## Abstract

Adhesion of antibiotic-resistant bacteria to medical device thermoplastics was evaluated by removal sampling at 1 and 24 hours. Vancomycin-resistant *Enterococcus faecalis* was removed in greater numbers than either methicillin-resistant *Staphylococcus aureus* or extended-spectrum beta-lactamase *Escherichia coli*, suggesting less adhesion and greater potential for transfer to patients wearing contaminated devices.

## Introduction

**Figure 1.**
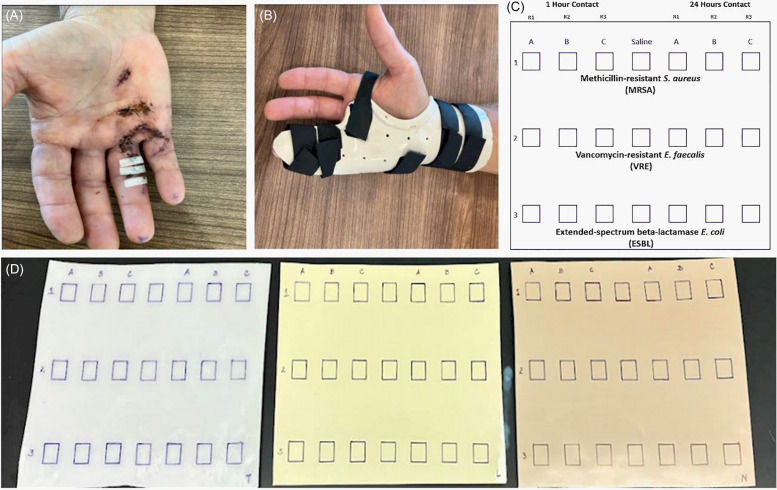
(A) Hand surgery incisions, (B) hand immobilized in a custom thermoplastic orthosis, (C) test material template, (D) thermoplastic sheets with bacterial inoculum sampling squares: Klarity ThermoSheets^TM^ | Klarity Contour Blend^TM^, NC | Spectrum^TM^ Beige (left to right).

Thermoplastic materials are routinely used to fabricate custom orthoses for patients with burns, surgical wounds, and traumatic injuries. These devices are worn for prolonged periods, handled repeatedly by healthcare providers and patients, and placed in close contact with compromised skin (Figure 1). Early investigations demonstrated that thermoplastic splints used in burn care become contaminated with bacteria, raising concerns regarding infection risk.^1,2^ Despite these observations, the incidence of pathogens, including antibiotic-resistant (AR) bacteria, on patient-used thermoplastics and their implications remains poorly understood.

Gram-positive organisms, particularly methicillin-resistant *Staphylococcus aureus* (MRSA) and vancomycin-resistant *Enterococcus* species, are frequently linked to healthcare-associated infections (HAIs). They readily colonize skin, persist on dry surfaces, and are readily transmitted via contact.^3–5^ These characteristics make gram-positve bacteria especially relevant to externally worn thermoplastic devices, which are repeatedly handled during removal and reapplication. In contrast, gram-negative bacteria are less frequently recovered from contaminated externally worn medical devices.^6^

Studies sampling either radiation therapy or orthotic immobilization thermoplastic devices demonstrated that these patient-worn devices harbor bacteria, including clinically relevant pathogens.^7–9^ However, considerably less is known about the potential of contaminated thermoplastics enabling bacterial transfer during routine handling or their persistence on these devices. Concerning transferability, assessing the degree of bacterial adhesion is essential for understanding infection risk and informing device hygiene practices. This pilot study evaluates the adhesive nature of gram-positive and gram-negative AR bacteria to patient-used thermoplastic appliances under controlled conditions. It represents the first known investigation of its type involving externally worn medical devices.

## Methods

Two unperforated thermoplastic materials commonly used in orthotic fabrication and one thermoplastic used in radiation therapy applications were obtained from commercial sources. Materials were processed using standard fabrication techniques and sectioned into marked sampling regions (Figure 1).^9^ Experimental procedures followed established methods previously used to assess bacterial attachment to different orthotic and radiation therapy thermoplastics.^7,9^

AR bacteria included MRSA, vancomycin-resistant *Enterococcus faecalis* (VRE), and extended-spectrum β-lactamase–producing *Escherichia coli* (ESBL). MRSA and VRE gram-positive cocci, ESBL, a gram-negative rod, were selected based on clinical relevance, survival on abiotic surfaces, and prominence in HAIs.^3–6^ Separate non-nutritive sterile saline suspensions were used to apply bacteria to each tested thermoplastic. This eliminated growth support and emphasized bacterial adhesion rather than replication.^6^

Inoculated thermoplastics were transferred to an ambient 35°C incubator. Thermoplastic sampling was performed at 1 and 24 hours to assess for changes in adhesion over time. Sufficient force was applied to the inoculated target squares during recovery sampling to mimic routine handling and/or wiping. Released bacteria were quantified using direct plate counts. GraphPad Prism software version 10.2 was used for statistical analysis.

This study was approved by the Institutional Biosafety Committee. Institutional Review Board approval was not required because no human or animal subjects were involved.

## Results

**Figure 2.**
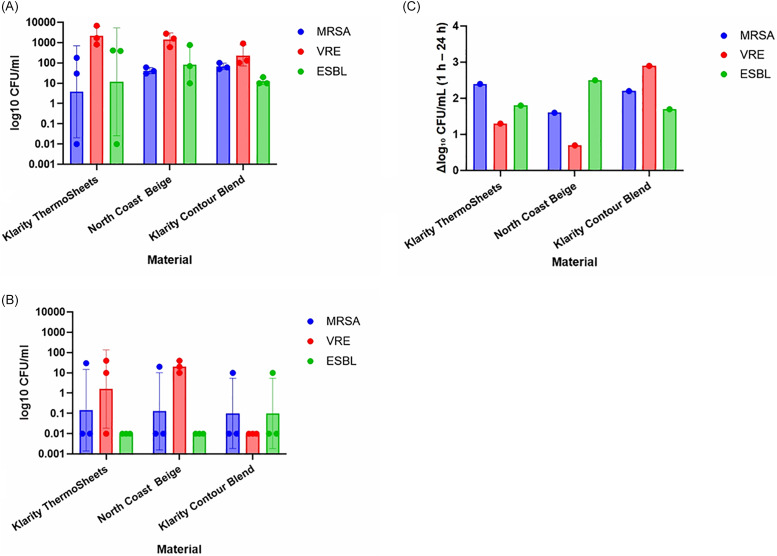
Two-way ANOVA of 1-hour log10 CFU data revealed a significant bacterium effect (*p* ≈ .02), while neither material (*p* ≈ .60) nor the interaction between bacterium and material (*P* ≈ .63) was considered significant. Panel C At 24 hours, no effects or interactions achieved significance of *P* = .05; bacterium *P* ≈ .10, material *P* ≈ .49, and interaction *P* ≈ .13. In all instances, fewer bacteria were recovered after 24 hours of contact than were recovered at 1 hour.

All three thermoplastics demonstrated AR bacteria removed at both 1 and 24-hour sampling intervals (Figure 2). At 1-hour removal sampling, VRE was removed in greater numbers than MRSA or ESBL across all thermoplastics. Although not statistically significant, similar patterns persisted. In all instances, decreased removal of ESBL, MRSA, and VRE was observed at the 24-hour sampling interval.

Additionally, the log_10_ difference (log Δ) in removed bacteria varied between thermoplastics: MRSA (∼2.4) for Klarity ThermoSheets^TM^, ESBL (∼2.5) for North Coast Beige^TM^, and VRE (∼2.9) for Klarity Contour Blend.

## Discussion

Neither Gram stain reaction nor thermoplastic type appears to have greatly influenced bacterial removal at 1 hour. However, VRE was removed in greater numbers than MRSA or ESBL from all thermoplastics, indicating its lower adhesion after 1 hour of contact. Fewer ESBL, MRSA, and VRE bacteria were removed from each material at 24-hour sampling, indicating increased adhesiveness. Differences in bacterial removal between thermoplastics likely reflect variations in physicochemical properties like surface topography and hydrophobicity. Regardless of stated differences, our results are consistent with earlier reports recovering AR bacteria by similar sampling of contaminated in-use patient thermoplastic orthoses and radiation therapy immobilization devices.^1,2,7^

Easier bacterial removal from contaminated devices suggests a greater transfer potential to patient skin during application, highlighting patient infection risk associated with improper handling and/or insufficient cleaning. Infection transmission occurs when bacteria are dislodged from contaminated devices transferring onto patient’s compromised skin and/or wounds. Contact-mediated transmission of gram-positive MRSA is frequently implicated in device-associated and skin-related infections.^3–5^

Conversely, decreased removal at 24 hours across all tested AR bacteria suggests increased surface adhesion even in nutrient-limited conditions. In such environments, bacteria attach and quickly increase their adhesiveness to underlying surfaces, a behavior consistent with the initial stages of biofilm development on medical devices.^6,10^ Adhesion therefore has important clinical implications. Increased material adhesion reduces the immediate-term transfer potential while enabling longer-term persistence on contaminated devices, increasing infection risk potential over time, particularly in the absence of routine cleaning.

Bacteria persisting in a dormant state, exhibiting strong adhesion, may remain viable on a contaminated device for extended periods. Conversely, loss of adhesion due to a lack of biofilm maintenance over time increases their probability of transferring to compromised tissue, resuming a vegetative (active) state now in a nutrient-rich environment.

## Clinical implications

Our results indicate thermoplastic devices can serve as potential reservoirs of AR bacteria and may facilitate transfer to patient skin. These observations are consistent with prior clinical studies reporting contamination of immobilizing thermoplastic devices.^1,2,7^

Our results further indicate that orthotic thermoplastics should be considered likely vehicles for bacterial transfer rather than inert materials. Gram-positive bacteria are particularly concerning due to their documented transfer from contaminated healthcare workers’ hands. Devices may be removed and reapplied multiple times per day, creating repeated opportunities for exogenous bacterial contamination.

These findings also highlight the need for standardized guidance on the hygiene of thermoplastic immobilization devices, particularly patient orthoses. Lack of routine cleaning allows bacterial persistence, thereby increasing transfer potential. Developing a clearer understanding of bacterial adhesion and persistence on thermoplastic medical devices will help inform cleaning recommendations.

This study was not intended to provide direct evidence linking tested AR bacteria to patient infections from wearing contaminated thermoplastic devices. Instead, it demonstrated that differences in bacterial adhesion to thermoplastics will influence the likelihood of bacterial transfer to patients.

## Limitations and future directions

This pilot study evaluated a limited number of bacteria and thermoplastics, and the findings should therefore be considered hypothesis-generating rather than broadly generalizable. The present work did not directly visualize transferred AR bacteria on thermoplastics. However, the observed difference in bacterial adhesion supports further investigation into early biofilm formation, since increased adhesion was suggested at 24 hours.

Future work will employ scanning electron microscopy to detect biofilm development and extracellular polymeric substance deposition on orthotic thermoplastics. The impact of substituting various body fluids (eg, perspiration, wound fluid) for sterile saline on bacterial adhesion and biofilm development is similarly planned. These studies will help clarify the conditions most likely to promote infection in patients wearing contaminated devices.

## Concluding views

This study demonstrated that AR bacteria readily transfer to orthotic thermoplastics and that bacterial adhesion changes over time under nutrient-deprived conditions. Reduced bacterial removal over time suggested increased adhesion, potentially reflecting early biofilm formation while sufficient stored energy reserves remained. These findings highlight the need for further investigation into bacterial adhesion and persistence on thermoplastic orthotic devices to better understand infection risk and inform prevention strategies in orthotic care.

## Data Availability

Study data sheets are available from the corresponding author upon reasonable request.
